# Modulation of Intestinal Epithelial Cell Proliferation, Migration, and Differentiation *In Vitro* by Astragalus Polysaccharides

**DOI:** 10.1371/journal.pone.0106674

**Published:** 2014-08-26

**Authors:** Chun Li Zhang, Hui Jun Ren, Meng Meng Liu, Xiao Gai Li, De Li Sun, Nan Li, Liang Ming

**Affiliations:** 1 Department of General Surgery, The People’s Hospital of Zhengzhou, Zhengzhou, China; 2 Department of Clinical Laboratory, The First Affiliated Hospital of Zhengzhou University, Zhengzhou, China; 3 Department of Pathology, The Third People’s Hospital of Zhengzhou, Zhengzhou, China; Aix-Marseille University, France

## Abstract

Previous studies have shown that Astragalus polysaccharides (APS) can be used to treat general gastrointestinal disturbances including intestinal mucosal injury. However, the mechanism by which APS mediate this effect is unclear. In the present study, the effects of APS on proliferation, migration, and differentiation of intestinal epithelial cells (IEC-6) were assessed using an in vitro wounding model and colorimetric thiazolyl blue (MTT) assays. The effect of APS on IEC-6 cell differentiation was observed using a light microscope and scanning electron microscope, and the expression of differentiation-specific markers of IEC-6 cells, such as cytokeratin 18 (CK18), alkaline phosphatase (ALP), tight junction protein ZO-2, and sucrase-isomaltase (SI), was determined by immunofluorescence assay (IFA) and real-time PCR. In addition, APS-induced signaling pathways in IEC-6 cells were characterized. Our results indicated that APS significantly enhance migration and proliferation of IEC-6 cells in vitro. APS-treated IEC-6 cells have numerous microvilli on their apical surface and also highly express CK18, ALP, ZO-2, and SI. Moreover, APS-treated IEC-6 cells, in which the activity and expression level of ornithine decarboxylase (ODC) were significantly elevated, also exhibited an increase in cellular putrescine, whereas no significant increase in TGF-β levels was observed. These findings suggest that APS may enhance intestinal epithelial cell proliferation, migration, and differentiation in vitro by stimulating ODC gene expression and activity and putrescine production, independent of TGF-β. Exogenous administration of APS may provide a new approach for modulating intestinal epithelial wound restitution in vivo.

## Introduction

The intestinal epithelium acts as a crucial barrier between the body and the luminal environment [1], [2]. Intestinal epithelial injury induced by a variety of noxious agents (chemical, physical, infectious, and inflammatory) may result in increased penetration and absorption of toxic substances, immunogenic responses, and disequilibrium of the host’s homeostasis. Thus, rapid resealing of the epithelial surface barrier following injuries is essential to preserving normal homeostasis. Studies have shown that intestinal epithelial wound healing and reestablishment of epithelial continuity can be achieved by cell replication within the crypts of Lieberkuhn and subsequent migration of the cells’ progeny onto the villus epithelium in the small intestine or onto the surface epithelium in the colon [3].

*Astragali Radix*, the root of *Astragalus membranaceus Bunge*, is a popular health-promoting herb that has been used for thousands of years in oriental medicine. Astragalus polysaccharides (APS) are the main ingredient of *Astragali Radix*. APS-I and APS-II are the major structural components of APS [4]. APS-I (molecular weight = 1,699,100 Da) consists of arabinose and glucose (molar ratio 1∶3.45), while APS-II (molecular weight = 1,197,600 Da) consists of rhamnose, arabinose, and glucose (molar ratio 1∶6.25∶17.86) [4], [5]. It is well documented that APS have been used as immune enhancers and display antibacterial, antiviral and antiparasitic activities [6]–[11]. In addition, APS have been used to treat hepatitis, diabetes, myocarditis, glomeruonephritis, tumors, and other illnesses with no obvious toxicity in clinical studies [12]–[15]. However, knowledge of how APS exert their bioactivities (e.g., anti-inflammatory and anti-tumor activities) is still limited.

As mentioned above, APS have a variety of biological activities and pharmacological properties, and previous studies have shown that APS can be used to treat general gastrointestinal disturbances including intestinal mucosal injury [16]. However, the mechanism by which APS mediate these effects is unclear. In our previous work, Astragalus Injection (AI) was found to have a protective effect in intestinal injury induced by lipopolysaccharide (LPS) in vitro (data not shown). *Astragali Radix* consists of many different components, including polysaccharides, glycosides, alkaloids, volatile oils, and organic acids. Although APS are the main component of *Astragali Radix*, whether APS play a key role in intestinal epithelial repair, and if so, the mechanism by which APS modulate epithelial cell proliferation and migration during normal turnover or intestinal wound repair, remains unknown. In the present study, we investigated the effects of APS on the proliferation, migration, and differentiation of normal rat small intestinal epithelial cells (IEC-6). The gene expression of ornithine decarboxylase (ODC) and transforming growth factor (TGF)-β, as well as ODC activity and putrescine production, were examined in IEC-6 cells. The present study will be helpful in elucidating the molecular mechanism underlying the modulation of intestinal epithelial restitution by APS and identify a potential novel approach for modulating intestinal epithelial wound healing in vivo.

## Materials and Methods

### Reagents and antibodies

APS isolated from 6-year-old *Astragalus membranaceus* samples with a purity of 98.5% were purchased from Dabang Animal Pharmaceutical Company (Inner Mongolia, China). Dulbecco’s modified Eagle’s medium (DMEM), dialyzed fetal bovine serum (FBS), Ethylene Diamine Tetraacetic Acid (EDTA), trypsin, propidium iodide (PI), Dulbecco’s PBS (D-PBS), gentamicin, 3-(4,5-dimethylthiazol-2-yl)-2,5-diphenyltetrazolium bromide (MTT), dimethyl sulfoxide, and sodium dodecyl sulfate (SDS) were obtained from Sigma (St. Louis, US). TRIzol reagent was obtained from Invitrogen, and the Oligotex mRNA Mini kits were obtained from Qiagen (Valencia, CA). Matrigel was purchased from BD Biosciences (San Diego, CA). The TGF-β ELISA kit and ornithine decarboxylase ELISA kit were obtained from R&D Systems (Minneapolis, MN). M-MLV reverse transcriptase was obtained from Promega (Madison WI). Rabbit polyclonal anti-cytokeratin was purchased from Santa Cruz. L-[1-^14^C]ornithine (specific activity, 52.1 Ci/mol) was purchased from NEN (Boston, MA).

### Cell culture

The rat small intestinal cell line IEC-6 (CRL 1592) was obtained from the American Type Culture Collection (Rockville, MD) at passage 13. The IEC-6 cells were maintained in T-150 flasks (Corning) in a humidified, 37°C incubator in an atmosphere of 90% air/10% CO_2_. The stock medium was DMEM containing 5% FBS, 2 mM L-glutamine, 50 µg/mL gentamicin, and 10 µg/mL insulin. The stock was passaged weekly at 1∶4 and fed three times per week. Passages 17–21 were used for the experiments. The cells were routinely checked for mycoplasma and always found to be negative.

### Cell proliferation assay

Cell growth was measured by MTT [3-(4,5-diethylthiazoly-2-yl)-2,5-diphenyltetrazolium bromide] assay [17]. Briefly, IEC-6 cells (5×10^2^ cells/well) were cultured in 96-well culture plates for 24 h. The growth medium was replaced with complete DMEM in the presence of different concentrations of APS (50, 100, 200, and 400 µg/mL). Control cells were fed with fresh medium without APS as well. After an additional 24 h in culture, the medium was removed and the cells were incubated with 20 µL of MTT solution (5 mg/mL) at 37°C for 4 h. MTT was then removed, and 200 µL of 100% dimethyl sulfoxide was added to each well. After 10 min, the absorbance of each well was measured with a microplate reader (Bio-Tek) at a wavelength of 570 nm. The viable cell number was proportional to the absorbance. All assays were performed in triplicate.

### Immunofluorescence assay (IFA)

IEC-6 cells were seeded on glass coverslips (in 6-well plates) at a density of 5×10^5^ cells per well with 200 µL of complete DMEM and grown under the conditions described above. After 24 h, 10 µL of APS was added to a final concentration of 200 µg/mL. The untreated IEC-6 cells were included as a control. The cells were harvested after 2∼4 days of incubation with APS, and IFA was performed as described previously [18]. The coverslips were removed and fixed with ice-cold acetone for 10 min. Following washes in phosphate-buffered saline (PBS), the cells were blocked with 5% normal goat serum in PBST (0.1% Triton-X100 in PBS) for 20 min. Then, the cells were incubated with rabbit polyclonal anti-cytokeratin 18 (1∶100) at 37°C for 1 h, followed by washing with PBS. The cells were incubated with FITC-conjugated goat anti-rabbit IgG (1∶100; Santa Cruz). Subsequently, the coverslips were mounted on glass slides and examined under a fluorescence microscope (Olympus).

### Scanning electron microscopy (SEM)

Cell cultures grown on glass coverslips were processed for SEM analysis as described by Stettler [19]. Briefly, cells that were cultured and treated as described for the IFA were fixed in 2.5% glutaraldehyde in 0.1 M phosphate buffer (pH 7.4) for 4 h at room temperature, followed by postfixation in 2% OsO_4_ in 0.1 M phosphate buffer for 1 h. The cells were then extensively washed in distilled water, dehydrated by passage through graded dilutions of ethanol and substituted with acetone. Cells were dried by sublimation in Peldri II (Plano GmbH, Marburg, Germany), sputter-coated with gold and observed by SEM (JSM-7500F, JEOL, Japan).

### Real-time PCR analysis

Transcription of the following six genes in the IEC-6 cells was evaluated by real-time PCR: cytokeratin 18 (CK18), alkaline phosphatase (ALP), tight junction protein ZO-2, sucrase-isomaltase (SI), ornithine decarboxylase (ODC), and transforming growth factor beta (TGF-β). G3PDH was used as a reference gene to normalize mRNA levels [20]. The primers were designed using the Primer5.0 software; details for gene-specific primers are listed in Table 1. IEC-6 cells were grown in DMEM containing 200 µg/mL APS for 1 or 4 d and then harvested for total RNA isolation using the TRIzol reagent. Total cDNA was generated using M-MLV reverse transcriptase and random primers and diluted at 1/30 with sterile water before use. Real-time PCR was performed in a total volume of 20 µL containing diluted cDNA (2 µL), 10 µL of 2× SYBR Premix Ex Taq (Takara, Japan), 0.4 µL of each primer (10 µM final concentration), 0.4 µL of 50× ROX Reference Dye II, and 6.8 µL of deionized water. PCR was run on an ABI 7500 fast real-time PCR system (Applied Biosystems, USA). The cycling conditions used were 95°C for 30 s, followed by 40 cycles of 95°C for 3 s and 60°C for 30 s. The specificity of amplicons was verified by melting curve analysis (60 to 95°C) after 40 cycles and agarose gel electrophoresis. A no-template control was included on each reaction plate. Relative expression levels of the target genes were normalized to G3PDH and then calculated using the comparative Ct (2^−△△Ct^) method [21]. Each sample had three replicates, and each experiment was repeated three times.

**Table 1:** Primers used in real-time PCR assays.

Gene	NCBI AccessionNumber	Primer sequence	Product size (bp)
Cytokeratin 18(CK18)	NM_053976.1	F 5′–CGCCAGACCCAGGAATACGA–3′	186
		R 5′–CACCACTTTGCCATCCACGAC–3′	
Alkaline phosphatase,intestinal (ALP)	NM_022665.3	F 5′–CCTTCACAGGGACCAAGTAAC–3′	187
		R 5′–TGGGGACAAGAGTCGGTAAT–3′	
Tight junctionprotein ZO-2 (ZO-2)	NM_053773.1	F 5′–AAGAAGAACATTCGCAAGAGC–3′	186
		R 5′–AGTTTGAAACAGGTCGGGTAG–3′	
Sucrase-isomaltase(EC 3.2.1.10)	NM_013061.1	F 5′–GGCACTACCTAACCATCCGC–3′	169
		R 5′–ATAAGTAGTGCAGGGCCCCA–3′	
Ornithinedecarboxylase (ODC)	J04791.1	F 5′–AGAGCACATCCAAAGGCAAAG–3′	198
		R 5′–CACGAAGGTCTCAGGGTCAGTA–3′	
Transforming growthfactor beta (TGF-β)	NM_053802.1	F 5′–GGGTTGGGATGTTAAGGG–3′	107
		R 5′–TCAGCGCCAAGATACGAC–3′	
G3PDH (Reference)	NM_022215.2	F 5′–GCCAAGATCGTGGGTAGTAATG–3′	127
		R 5′–CATTCTCGTGTTGCGTGTTGA–3′	

### Measurement of cell migration

The cell migration assay was performed as previously described [22]. Briefly, the cells were plated at 6.25×10^4^/cm^2^ in complete DMEM on 35-mm dishes thinly coated with Matrigel according to the manufacturer’s instructions. The cells were fed on day 2, and migration was tested on day 4. To initiate migration, a portion of the cell layer was removed by scratching with a single-edge razor blade cut to ∼27 mm in length. The scratch began at the diameter of the dish and extended over an area 7–10 mm wide. The medium was changed to remove floating or damaged cells, and 100 µL of APS was added to final concentrations of 50, 100, 200, and 400 µg/mL. The cells were returned to the incubator for 24 h, during which the cells migrated over the denuded area. Cell migration was observed at 100× magnification using a phase-contrast inverted microscope with a Polaroid camera. Data collection and image analysis were completed with NIH Image software. The results are reported as the number of migrating cells per millimeter of scratch (cells/mm). All experiments were performed in triplicate.

### ODC activity assay

ODC activity in IEC-6 cells was assayed with a radiometric technique in which the amount of ^14^CO_2_ liberated from DL-[1-^14^C]ornithine was estimated [23]. Briefly, after experimental treatment, the plates were placed on ice; the monolayers were washed three times with cold Dulbecco’s PBS (DPBS), and 0.5 mL of 1 mM Tris buffer (pH 7.4) containing 1 mM EDTA, 0.05 mM pyridoxal phosphate, and 5 mM dithiothreitol was added. The cells were frozen and thawed three times, scraped into Eppendorf tubes, sonicated, and centrifuged at 18,000×g for 30 min at 4°C. The ODC activity of an aliquot of the supernatant was incubated in a stoppered tube in the presence of 6.8 nmol of [^14^C]ornithine for 15 min at 37°C. The ^14^CO_2_ liberated by the decarboxylation of ornithine was trapped on a piece of filter paper impregnated with 20 µL of 2 N NaOH, which was suspended in a center well above the reaction mixture. The reaction was stopped by the addition of trichloroacetic acid to a final concentration of 5%. The ^14^CO_2_ trapped in the filter paper was measured by liquid scintillation spectroscopy. Aliquots of the supernatant were assayed for total protein using the method described by Bradford [24]. Enzymatic activity is expressed as nmol of CO_2_/mg protein/h.

### Cellular putrescine analysis

Cells were cultured and treated as described for the cell proliferation assay. The cellular putrescine content was analyzed by HPLC as previously described [22]. After washing the cell monolayers with cold DPBS, 0.5 mL of 0.5 M perchloric acid was added to the plates and the cells were frozen at −80°C until for the time of extraction, dansylation, and HPLC. The standard curve encompassed the range from 0.31 to 10 µM. Putrescine content is expressed as nanomoles per milligram of protein.

### ELISA

TGF-β proteins in cell culture supernatants and ODC proteins in the cell lysates were determined using ELISA kits according to the manufacturer’s instructions. Samples for TGF-β detection were activated by acidification with 1 N HCl for 60 min to a pH of 2.5, followed by neutralization with 1 N NaOH. The cells for ODC detection were sonicated for 30 sec on ice, centrifuged for 5 min at 1,000×g in a chilled microfuge, and the supernatant thus obtained was used as cell lysates. The major steps of ELISA were shown as follows. Samples were added to 96-well plates precoated with anti-TGF-β or anti-ODC monoclonal antibodies that bind soluble TGF-β or ODC in solution. After several incubation steps, washing procedures, and a final incubation with a chromogenic substrate, the absorbance at 492 nm was measured with a microplate reader (Bio-Rad). All samples were tested in triplicate.

### Statistical analysis

All data are expressed as the means ± standard deviation (SD). Inter-group statistical analyses were performed with one-way factorial ANOVA (LSD test) using SPSS version 17.0 software (SPSS Inc., Chicago, IL). Differences were considered statistically significant at *P*<0.05.

## Results

### Effect of APS on IEC-6 cell proliferation

**Figure 1:**
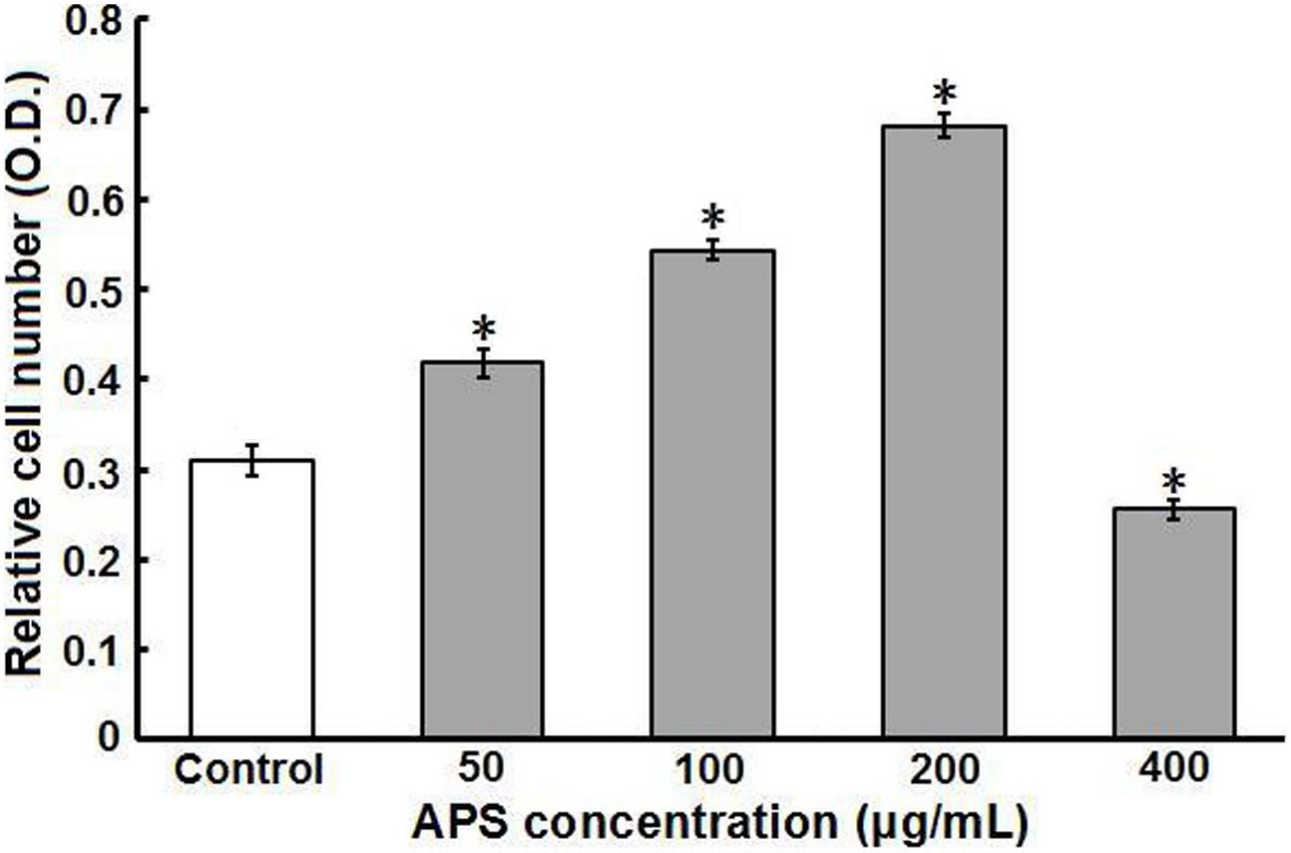
Effect of APS on intestinal epithelial cell (IEC-6) proliferation in vitro. IEC-6 cells were cultured in the presence of 50–400 µg/mL APS for 24 h, and cell proliferation was assessed by MTT assays as described in “Materials and Methods”. The data are expressed as the means ± SD of absorbance values from 3 separate experiments. *P<0.01 compared to control (untreated IEC-6 cells).

IEC-6 cells were grown in complete DMEM in the presence or absence of APS for 24 h. Cell proliferation was measured by the MTT assay. As shown in Figure 1, APS significantly increased IEC-6 cell proliferation in vitro in a dose-dependent manner (50–200 µg/mL) compared with the untreated control (*P*<0.01). After 200 µg/mL APS was added to the medium, the cell proliferation rate was increased 2.2-fold over the control level. On the other hand, a higher concentration of APS (400 µg/mL) inhibited IEC-6 cell proliferation compared with the control (*P*<0.01). Additionally, when IEC-6 cells pretreated for 48 h with 200 µg/mL APS were treated with 500 µmol/L hydrogen peroxide (H_2_O_2_) for 40 min, the inhibition of IEC-6 proliferation caused by H_2_O_2_ was significantly lowered compared with that in controls, which were not pretreated with APS (data not shown).

### Cell migration

**Figure 2:**
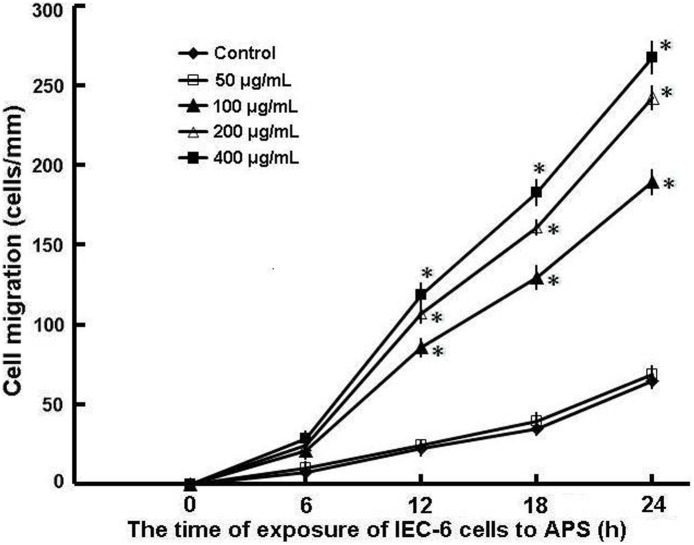
Effect of APS on intestinal epithelial cell (IEC-6) migration in vitro. Standard wounds were made with razor blades in confluent monolayers of IEC-6 cells as described in “Materials and Methods”. Wounded monolayers were incubated with different concentrations of APS for 24 h. Then, the cells that migrated over the wounded edge were counted at 0 h, 6 h, 12 h, 18 h, and 24 h after wounding. The data are expressed as the means ± SD of 12 observations. *P<0.01 compared to control (untreated IEC-6 cells).

The addition of APS (100–400 µg/mL) to complete DMEM significantly enhanced IEC-6 cell migration over the wounded edge compared with migration of the untreated IEC-6 cells after wounding in a model that mimics the early stage of epithelial restitution in vitro (*P*<0.01). Cell migration into the wound area was observed as early as 4 h after the addition of APS to the wounded monolayers. Significant differences were observed in high APS concentration groups (200 and 400 µg/mL) at 8 h (data not shown), in all treated groups at 12 h, and were maximal at 24 h. Enhancement of IEC-6 cell migration (restitution) by APS was dose-dependent, reaching a maximum at the 400 µg/mL concentration of APS (Figure 2). The number of cells migrating over the wounded edge in IEC-6 cells treated with 400 µg/mL APS was almost four times that of the untreated cells at 24 h after wounding (65±5 cells/mm in the untreated cells, 267±11 cells/mm in APS-treated cells, n = 12, *P*<0.01). There were no significant differences in the cell migration rates at 24 h between the untreated cells (65±5 cells/mm, n = 12) and the cells treated with 50 µg/mL APS (68±6 cells/mm, n = 12) (*P*>0.05).

### Effect of APS on IEC-6 cell morphology

**Figure 3:**
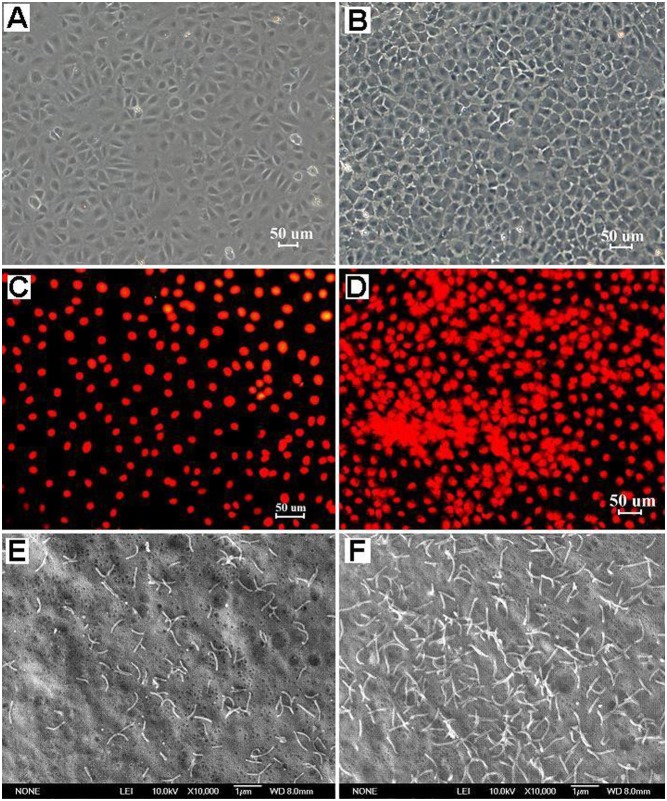
Morphological changes of APS-treated IEC-6 cells. Normal, untreated IEC-6 cells formed a simple monolayer of flat epithelial cells and were in an irregular arrangement with an immature shape (A). The untreated IEC-6 cells contained large, oval nuclei that exhibited red fluorescence by propidium iodide staining (C) and showed few microvilli on the apical surface under a scanning electron microscope (E). IEC-6 cells treated with APS (200 µg/mL) for 4 d were more closely packed together and showed many pseudopodia establishing intercellular contacts (B). The nuclei of APS-treated cells were arranged regularly in rows or clusters, implying those cells exhibited a tendency towards gland formation (D). The scanning electron micrograph showed numerous long, slender microvilli distributed over the apical pole of treated cells (F).

The IEC-6 cell line consists of a homogeneous population of flat, epithelial-like cells with large, oval nuclei that grow as colonies of polygonal, closely opposed cells (Figure 3, A and C). However, four days after treatment with APS, IEC-6 cells with small nuclei were more closely packed together, and the cell-cell boundaries were more clearly visible (Figure 3B). Many pseudopodia extended from the cell borders to neighboring cells, establishing intercellular contacts (Figure 3B). As indicated in Figure 3D, which shows the propidium iodide-stained nuclei of treated IEC-6 cells, some of these cells were arranged in rows or clusters, which exhibits a tendency towards gland formation. Meanwhile, SEM showed that numerous long, slender microvilli were distributed over the apical pole of treated IEC-6 cells (Figure 3F). However, fewer microvilli were localized on the surface of untreated IEC-6 cells (Figure 3E).

**Figure 4:**
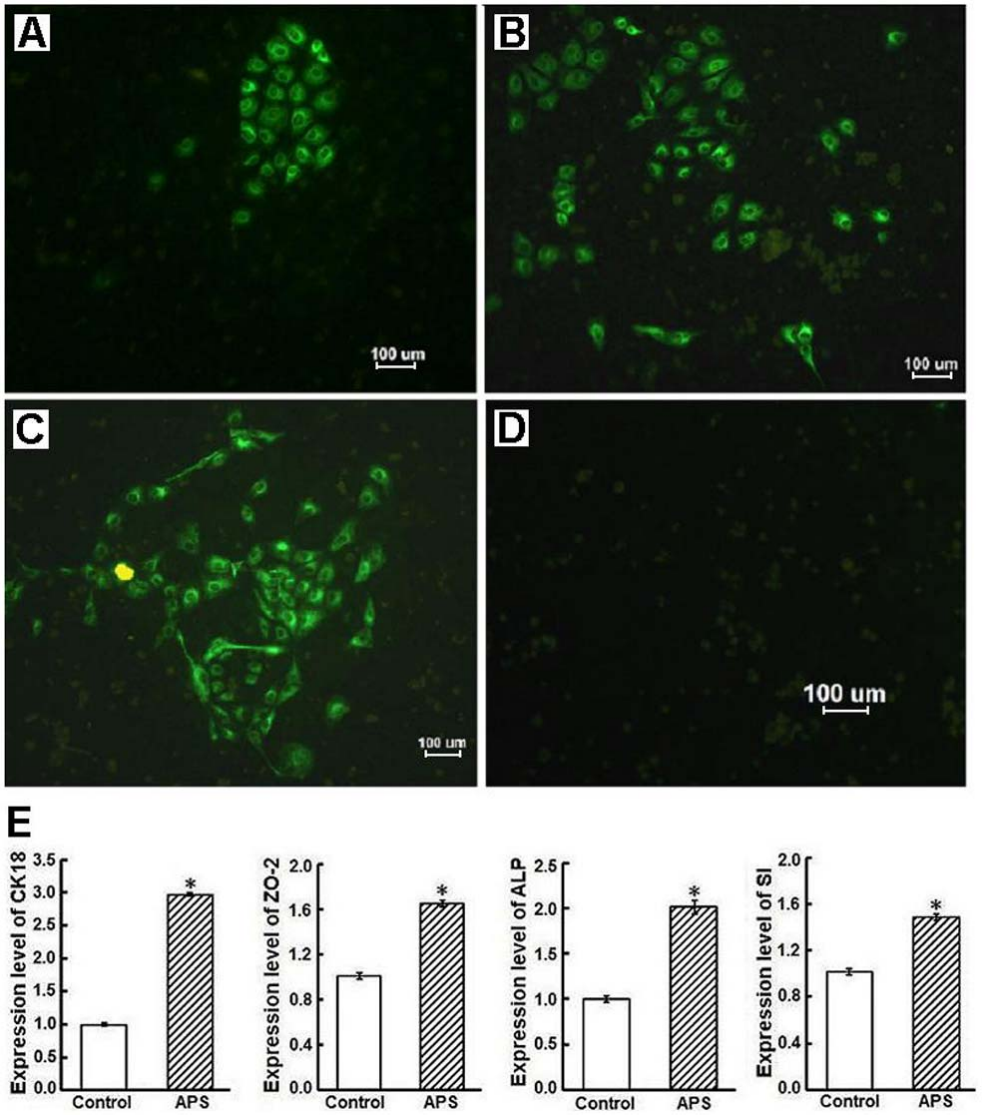
APS-treated IEC-6 cells expressed multiple molecular markers of epithelial differentiation. (A, B, and C) Immunofluorescence assay (IFA) showed that cytokeratin 18 (CK18) was clearly detected in the cytoplasm of APS-treated IEC-6 cells with a green color. The CK18-positive cells increased after 2 to 4 days of incubation with APS. (D) No fluorescence was found in the cytoplasm of untreated cells. (E) Relative expression of the four genes (CK18, ZO-2, ALP, and SI) in APS-treated cells was analyzed by real-time PCR. Expression levels were normalized to the G3PDH gene and are presented as the expression relative to that of the controls (mean ± SD, n = 9). Controls are arbitrarily assigned a value of 1. *P<0.01 compared to control (untreated IEC-6 cells).

In addition, cytokeratins as markers of epithelial differentiation were clearly detected by IFA in the cytoplasm of the APS-treated cells. The CK18-positive cells were in cluster or sporadically distributed among the CK18-negative cells. The number of positively stained cells increased during 4 days of incubation with APS (Figure 4A, 4B, and 4C), and the rates of CK18-positive cells increased from 5% to 68%. However, no fluorescence was always observed in the untreated cells (Figure 4D). Furthermore, the cells treated with APS (200 µg/mL) for 4 d also exhibited multiple molecular characteristics of intestinal epithelial differentiation. Real-time PCR analysis revealed that the transcription levels of mRNA (CK18, ALP, ZO-2, and SI) were obviously increased in the treated cells compared with the control cells (*P*<0.01) (Figure 4E).

### Effect of APS on ODC and putrescine in IEC-6 cells

**Figure 5:**
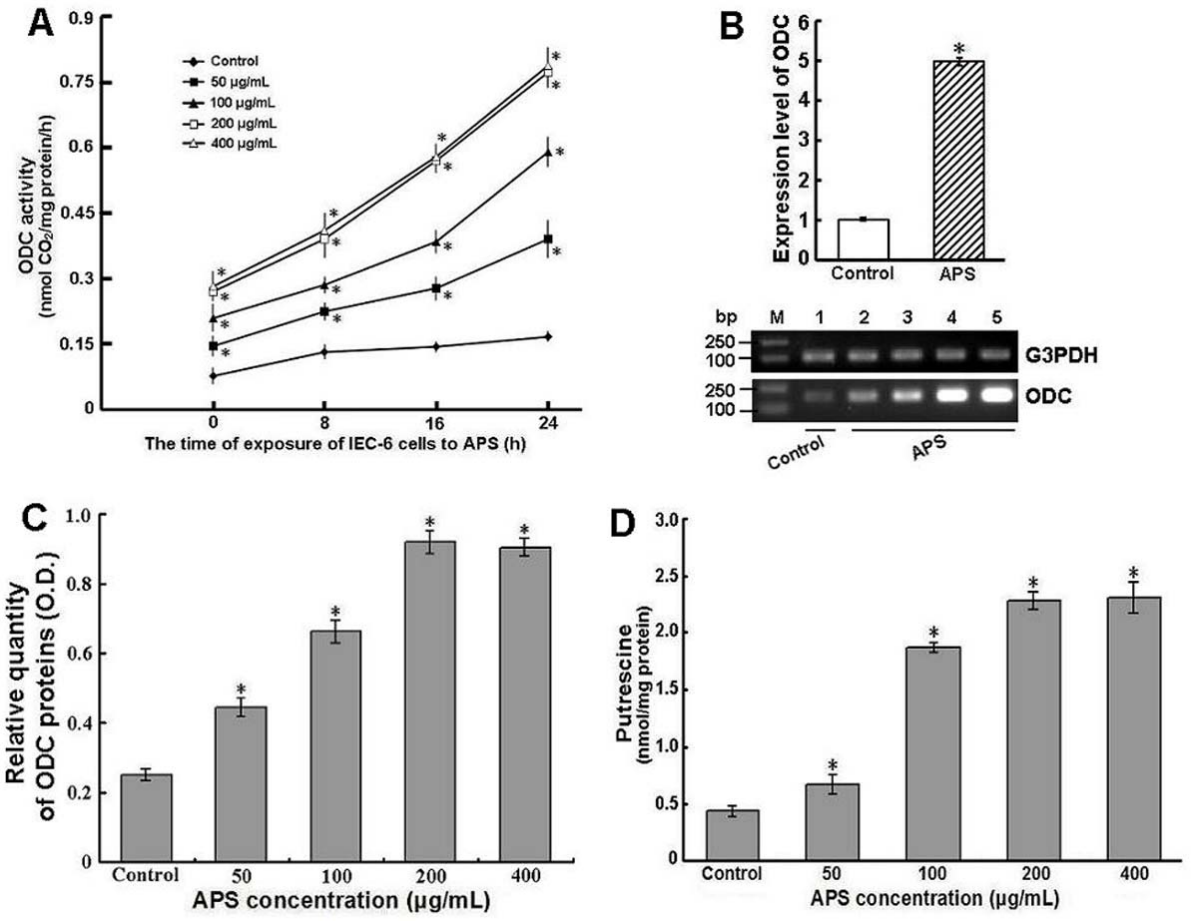
Effects of APS on ODC and polyamine in IEC-6 cells. IEC-6 cells were cultured in complete DMEM in the presence of APS (50, 100, 200, and 400 µg/mL, final concentrations) for 24 h. Then, cells were used to analyze ODC activity (A), protein levels (C) and putrescine levels (D) as described in “Materials and Methods”. (B) The ODC gene expression in IEC-6 cells treated with 200 µg/mL APS was analyzed using real-time PCR. Expression levels were normalized to the G3PDH gene and are presented as the expression relative to that of the controls (mean ± SD, n = 9). Controls are arbitrarily assigned a value of 1. *P<0.01 compared to control (untreated IEC-6 cells). The real-time PCR products were confirmed by agarose gel electrophoresis (lane M: DNA size markers; lane 1: control cells; lane 2: 50 µg/mL; lane 3: 100 µg/mL; lane 4: 200 µg/mL; lane 5: 400 µg/mL).

IEC-6 cells were cultured in complete DMEM containing different concentrations of APS (50–400 µg/mL). The ODC enzyme activity level was determined in IEC-6 cells at 0, 8, 16, and 24 h (Figure 5A). ODC activity was significantly increased in treated cells with increased APS concentrations (50–400 µg/mL) compared with that in untreated cells (*P*<0.01). When IEC-6 cells were exposed to 200 and 400 µg/mL of APS for 24 h, the ODC activity level was 4.6-fold and 4.7-fold greater than that of untreated cells, respectively (*P*<0.01). However, there was no significant difference between the two groups (*P*>0.05).

ODC proteins in cell lysates were measured by ELISA 24 hours after incubation with APS. The results showed that ODC proteins increased in APS-treated cells in a dose-dependent manner (50–200 µg/mL), and no significant difference was found in ODC protein expression between the concentrations of 200 and 400 µg/mL (Figure 5C). In addition, following administration of 200 µg/mL APS, the ODC mRNA level detected by real-time PCR analysis was increased approximately 4.9-fold after 24 h compared with untreated cells (*P*<0.01) (Figure 5B). A higher increase in the ODC mRNA level was detectable after 96 h in treated cells with differentiation characteristics (data not shown).

APS-treated IEC-6 cells with high ODC enzyme activity also exhibited increased amounts of cellular putrescine (Figure 5D). A 1.5- to 5.3-fold increase in putrescine was observed in IEC-6 cells exposed to different concentrations of APS for 24 h compared to control cells (*P*<0.01).

### TGF-β assay

**Figure 6:**
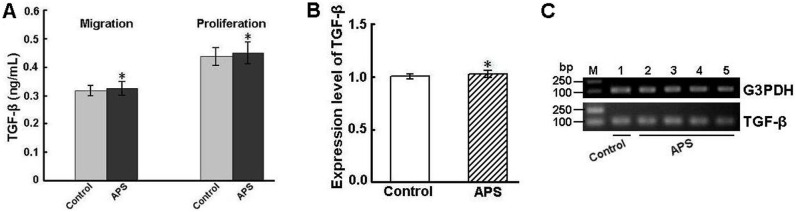
TGF-β production by APS-treated IEC-6 cells. (A) TGF-β levels were measured in the supernatants of IEC-6 cells treated with 200 µg/mL APS as described in “Materials and Methods”. Supernatants were obtained after a 24-h incubation period from migration and proliferation studies. Data are expressed as the means ± SD of three separate experiments. (B) IEC-6 cells were obtained from the migration study after 24 h of treatment with 200 µg/mL APS. The level of TGF-β transcripts in treated cells was analyzed using real-time PCR. Expression levels were normalized to the G3PDH gene and are presented as the expression relative to that of the controls (mean ± SD, n = 9). Controls are arbitrarily assigned a value of 1. *P>0.05 compared to control (untreated IEC-6 cells). (C) The real-time PCR products were confirmed by agarose gel electrophoresis (lane M: DNA size markers; lane 1: control cells; lane 2: 50 µg/mL; lane 3: 100 µg/mL; lane 4: 200 µg/mL; lane 5: 400 µg/mL).

To assess whether APS may modulate intestinal epithelial cell restitution through a TGF-β-dependent pathway, culture supernatants of APS-treated IEC-6 cells were tested for TGF-β production by ELISA. As shown in Figure 6A, the TGF-β level was not significantly increased in supernatants of the wounded and APS-treated monolayers compared with that of the controls (*P*>0.05). Similar results were obtained in the proliferation assays.

Meanwhile, in the migration assays, real-time PCR showed that there was no significant difference in the TGF-β mRNA level between the APS-treated monolayers and the controls (Figure 6, B and C).

## Discussion

Intestinal barrier function is based on the physiological renewal or pathological repair of intestinal epithelia. The continuity of the epithelial surface is reestablished by at least three distinct mechanisms after extensive destruction [25]. First, epithelial cells adjacent to the injured surface migrate into the wound to cover the denuded area. Second, the proliferation of epithelial cells is necessary to replenish the decreased cell pool. Third, maturation and differentiation of undifferentiated epithelial cells is needed to maintain the numerous functional activities of the mucosal epithelium. The separation of intestinal epithelial wound healing into three distinct processes is simplified and artificial. These three wound-healing processes overlap, and distinct processes may not be observed in vivo. However, this simplified and artificial model may provide a tool to better understand the physiology and pathophysiology of intestinal epithelial wound healing. Thus, the effects of APS on intestinal epithelial cell proliferation, migration, and differentiation were observed separately in vitro in the present study.

IEC-6 cells were derived from normal neonatal rat small intestine and have characteristics of crypt-type epithelial cells. The cells are non-tumorigenic and retain the undifferentiated character of epithelial stem cells. These cells exhibit a number of features similar to normal cells in culture: i.e., a normal rat diploid karyotype, strong density inhibition of growth, lack of growth in soft agar, and a low plating efficiency when seeded at low density [26]. The establishment of IEC-6 cell lines plays an important role in functional research on small intestine epithelial cells such as growth, differentiation, metabolism, pharmacological effects, and the pathophysiological changes and mechanisms in the intestinal mucosa resulting from various pathogenic factors. Moreover, this cell line has been broadly used in studies on cellular, molecular and genetic mechanisms of small intestinal mucosal repair [27]–[29].

In the present study, the MTT assay was used to determine whether the promotion of cell proliferation was a peculiarity of APS. The results showed that APS significantly promoted IEC-6 cell proliferation in vitro in a dose-dependent manner (50–200 µg/mL) whether the cells were normal or injured by H_2_O_2_. However, a higher concentration of APS (400 µg/mL) inhibited IEC-6 cell proliferation. These results suggest that APS may regulate IEC-6 cell proliferation from two sides. Moreover, previous studies have shown that low-dose APS can enhance immune function, while high-dose APS have the opposite effect [14]. Thus, the optimal dose range of APS should be determined for applications in accelerating intestinal epithelium restitution in vivo.

ODC, the first and most likely the rate-limiting enzyme in the polyamine biosynthetic pathway, is intimately associated with cell proliferation and function [23], [30], [31]. ODC has been shown to be crucial to cell growth by experimental approaches that interfere with the enzyme’s function. For example, inhibition of ODC activity by the specific inhibitor difluoromethylornithine arrests the growth of cultured cells in vitro [32]. ODC shows low expression in normal IECs but exhibits high levels of activity in IECs with high rates of proliferation and migration [33]. The current study clearly showed that APS-treated IEC-6 cells displayed elevated ODC activity and expression levels compared to control cells. The increases in ODC activity and expression levels reached a maximum in IEC-6 cells treated with 200 µg/mL APS, which is consistent with the results of the cell proliferation assay. However, the ODC activity levels could not be further increased in IEC-6 cells treated with 400 µg/mL APS, which was similar to that in cells exposed to 200 µg/mL APS. The explanation for the opposite effects of 400 µg/mL APS on cell proliferation and ODC activity is not clear. Moreover, the ODC mRNA level was increased in APS-treated IEC-6 cells from the cell migration assay in a dose-dependent manner (data not shown) and more highly increased in APS-treated cells with differentiation features. These results indicated that APS may promote IEC proliferation and migration by stimulating ODC gene expression and activity.

This early, rapid reepithelialization is a primary repair modality in the gastrointestinal tract and requires cellular polyamines [25]. Increasing evidence has demonstrated that the cellular polyamines spermidine and spermine and their precursor putrescine participate in the regulation of mucosal restitution and are essential for maintaining gastrointestinal mucosal integrity [34]. Cellular polyamine regulation is the point of central convergence for the multiple signaling pathways driving epithelial cell motility, proliferation, and differentiation [35]. The formation of putrescine from ornithine is catalyzed by ODC. Our data showed that when IEC-6 cells were treated with APS, cellular putrescine levels increased significantly with increasing APS concentrations. This result was consistent with the increase in ODC activity, indicating that the increases of ODC activity and cellular putrescine by APS may promote IEC growth, proliferation, and migration. These findings suggest that polyamine biosynthesis pathway may play an important role in modulation of intestinal epithelial wound restitution by APS. Certainly, future experiments will be necessary to determine the changes of other enzymes and metabolites involved in polyamine biosynthesis pathway.

Our results showed that significant morphological changes were observed in APS-treated IEC-6 cells in comparison with control cells. The treated cells formed a regular arrangement. Typically differentiated cells had an abundance of cytoplasm and a small nucleus. Numerous microvilli were distributed over the apical surface, however, only few microvilli were observed on the surface of control cells. The absence of a multilayer structure indicated that these treated cells did not lose their contact inhibition characteristics. The existence of pseudopodia extending from the cell borders to neighboring cells is also a typical feature of terminally differentiated enterocytes [36]. Meanwhile, APS-treated IEC-6 cells highly expressed many specific markers of epithelial differentiation, such as cytokeratin 18, alkaline phosphatase, tight junction protein ZO-2, and sucrase-isomaltase. These results indicated that these treated cells had differentiation characteristics of functional enterocytes.

Intestinal wound healing is initiated by the migration of epithelial cells found adjacent to the injured surface into the wound to cover the denuded area. This epithelial restitution occurs within a period of minutes to hours and does not require cell proliferation. Although the exact mechanism of restitution has not been elucidated, TGF-β has been shown to play a key role in regulating epithelial cell migration and wound repair. Some studies also demonstrated that IEC-6 migration by interleukin-1β, interferon-γ, epidermal growth factor, and TGF-α is TGF-β-dependent [37], [38]. However, in other studies, the intestinal epithelial cell restitution was independent of TGF-β [39]. Our studies found that there was no significant change in the expression level of TGF-β in APS-treated IEC-6 cells after wounding. This result suggests that the mechanism by which APS increases IEC-6 cell migration possibly through a TGF-β-independent pathway. An increase of TGF-β production stimulated by a natural stimulus of the wound healing process can promote IEC-6 cell migration, but at the same time suppress intestinal epithelial cell proliferation [40]. Because of having a strong effect on cell proliferation, the addition of APS may suppress the increase in TGF-β expression, which leads to no change in TGF-β production. Moreover, whether APS as immune modulators also play a role in suppressing the expression of TGF-β is still unclear. Hence, these speculations need to be verified in further experiments.

In summary, the present study demonstrated that APS accelerate intestinal wound repair by stimulating IEC proliferation, migration, and differentiation, elevated ODC activity and expression level and putrescine production, and had no effects on TGF-β levels. APS may have the potential benefit of acting as intestinal epithelial wound healing modulators in vivo.
